# Designing a Formulation of the Nootropic Drug Aniracetam Using 2-Hydroxypropyl-β-Cyclodextrin Suitable for Parenteral Administration

**DOI:** 10.3390/pharmaceutics10040240

**Published:** 2018-11-18

**Authors:** Sebastian D. Goldsmith, Arlene McDowell

**Affiliations:** School of Pharmacy, University of Otago, Dunedin 9054, New Zealand; sebgsmith@hotmail.com

**Keywords:** aniracetam, 2-hydroxypropyl-β-cyclodextrin, parenteral, solubility, inclusion complex

## Abstract

The nootropic drug aniracetam is greatly limited in its application by low aqueous solubility and a poor oral bioavailability. The primary aim of this study was to design a parenteral formulation of aniracetam that can be administered intravenously. Complexation of aniracetam with 2-hydroxypropyl-β-cyclodextrin (HP-β-CD) was investigated as a strategy to enhance solubility. A phase solubility analysis was performed to quantify the extent of improvement. An 819% increase in the solubility of aniracetam was obtained, reaching 36.44 mg/mL. This marked increase enables aniracetam to exist in an aqueous solvent at levels sufficient for parenteral dosing. A stability test was then devised using a design of experiment approach. The aniracetam-HP-β-CD formulation was subjected to different relative humidity and temperature and cyclodextrin concentrations over a 12-week period. Key changes in FTIR vibrational frequencies suggest the benzene moiety of aniracetam was introduced into the hydrophobic cavity of HP-β-CD. These results are highly supportive of the formation of a predictable 1:1 molar stoichiometric inclusion complex, explaining the improvement seen in physiochemical properties of aniracetam following formulation with HP-β-CD. This novel formulation of aniracetam suitable for parenteral administration will have utility in future studies to further elucidate the pharmacokinetics of this drug.

## 1. Introduction

There is growing interest in the use of memory enhancing drugs to alleviate memory impairments that accompany aging, disease, and injury. Age-related and neurodegenerative brain damage is increasing in prevalence [1], and the need to treat conditions such as Parkinson’s and Alzheimer’s disease, which confer cognitive impairment, has become more critical due to the growing elderly demographic. Consequently, the endeavor to search for compounds that can effectively ameliorate damage to cognitive functions has gained recent momentum.

Aniracetam is one of the more popular compounds of interest for cognitive decline, one that is presumed to act as a memory enhancer [2]. Aniracetam (*N*-anisoyl-2-pyrrolidinone) is an analogue of piracetam [2], and belongs to a group of cognition enhancers called nootropic drugs [3]. Nootropics describe a class of psychoactive drugs that both directly and selectively improve the efficiency of higher telencephalic integrated activities. Characteristic clinical effects of a nootropic include enhancements in learning acquisition and memory, whilst improving the brain’s general resistance to impairing agents [4]. The current consensus is that aniracetam works through a glutamatergic mechanism of action [5], as a positive allosteric modulator on the AMPA subtype [6]. Aniracetam slows both the deactivation and desensitization of AMPA receptors via stabilizing the glutamate bound conformation, altering ion flux [7]. Depending on the conformational composition on the inotropic receptors, there are different actions on higher telencephalon integrated activities [8,9]. Consequently, modulation of these receptors by aniracetam is speculated to explain the altered cognition and memory improvements observed in animal models [7].

Limited studies exist on aniracetam and its memory enhancing capability, and those that do exist present conflicting evidence. Aniracetam (25–100 mg/kg) restored object recognition in rats impaired by age, scopolamine, and nucleus basalis lesions [10]. Oral doses of aniracetam (10–100 mg/kg) have also led to improved learning acquisitions and prevented induced short-term amnesia in rodents with impaired learning or memory [11]. Similarly, aniracetam improved memory and cognitive functionality whilst exerting a favorable effect on emotional stability for human patients with dementia [12]. Aniracetam also improved pyscho-behavioural test results of memory and emotional stability following administration in elderly patients suffering from dementia of the Alzheimer’s type [13]. It is important to note that these studies only tested for cognition enhancement in cases where the subjects had a pre-existing impairment. Minimal evidence exists to suggest that the cognition enhancing effects of aniracetam work in patients with normal cognitive function. This may explain why there is a notable discrepancy between the therapeutic potential of aniracetam and the prediction models derived from research in normal, healthy subjects [2]. Only one study has investigated the therapeutic efficacy of aniracetam in normal healthy mice, exploring whether the drug may have potential therapeutic applications for individuals lacking cognitive deficits [14]. Healthy mice were tested on a variety of aspects of cognitive behavior including learning acquisitions, fear conditioning, and spatial learning. A daily oral dose of 50 mg/kg of aniracetam did not improve cognitive performance or alter the behavior of the healthy mice [14]. This is consistent with earlier studies in monkeys and pigeons, where memory was not enhanced with aniracetam [15].

The low aqueous solubility of aniracetam (0.147 mg/mL at pH 1.2 and 0.138 at pH 7.5) [16] and a low oral bioavailability of 0.2% has greatly limited the clinical utility of this compound [17]. In addition, aniracetam is extensively metabolized during first-pass metabolism in the liver, and extensively hydrolyzed in the gastrointestinal tract [16]. These features prove problematic, as systemic concentrations of aniracetam become difficult to detect and are time dependent. As a result, large gaps in the pharmacology of aniracetam exist, restricting further development towards clinical use.

Improving aqueous drug solubility can be achieved by using cyclodextrins through the formation of a non-covalent, inclusion complexes [18,19]. The torus structure of a cyclodextrin allows it to act as a host, harboring hydrophobic drugs in its non-polar cavity whilst existing in an aqueous environment due to its hydrophilic exterior [20], and so, act as water-soluble drug carriers suitable for injection [18]. In addition to their solubility enhancing effects, cyclodextrins have also been reported to improve the bioavailability and stability of susceptible drugs with minimal toxicity and irritation [21,22], with 2-hydroxylpropyl-β-cyclodextrin (HP-β-CD) being favored [23]. There is one account of aniracetam being incorporated into a 2,6-dimethyl-β-cyclodextrin [24]. However, the study was not based on the premise to investigate subsequent formulation design [24]. There were no records of a phase solubility profile or methods of incorporation used in the study, failing to confirm whether or not the formulation used contained precipitates. No data exists on whether the inclusion complex used enhanced the solubility of aniracetam.

A formulation that can be administered intravenously is essential to characterize the pharmacokinetics and subsequent clinical effect of aniracetam. Consequently, the aim of this study is to investigate the formulation strategy of HP-β-CD to enhance the solubility of aniracetam and contribute to developing a parenteral formulation to overcome existing bioavailability limitations.

## 2. Materials and Methods

### 2.1. Materials

Aniracetam powder (99%, CAS#72432-10-1) was obtained as a gift sample from M. Colombo, Department of Psychology, University of Otago. HP-β-CD (Kleptose^®^ HPB, MW 1440) was obtained from a gift from Roquette Lestrem, France. Methanol (99.8%) and acetonitrile (99.9%) were purchased from Merck (Auckland, New Zealand); formic acid (98%) was obtained from Sigma Aldrich, New Zealand. All other regents were of analytical grade. Purified water (Milli-Q Purification System, Auckland, New Zealand) was used throughout these studies.

### 2.2. Phase Solubility Analysis

The phase solubility analysis was carried out according to the method described in [25]. Aliquots (50, 200, 500, 800, and 1000 μL) of HP-β-CD solution (50% *w*/*v*) were added to constant amounts of aniracetam (100 mg) in sample tubes, and made up to 1 mL with purified water. Each concentration was carried out in triplicate. The sample mixtures were then shaken in an orbital mixer incubator (Ratek, Newcastle, Australia) at 30 °C for 24 h. Samples were then centrifuged at 9218 RPM for 1 min. From each sample, 200 μL aliquots of the resultant supernatant were extracted and diluted 1:1000 with the mobile phase (50:50 acetonitrile:water with 0.1% formic acid) before being analyzed by HPLC. The apparent solubility of aniracetam was calculated, and the stability constant, *Kc*, was calculated according to the Higuchi-Connors equation (Equation (1)).

(1) Kc=slope/(Intercept×(1−slope))

### 2.3. Quantification of Aniracetam

Reverse phase high pressure liquid chromatography (RP-HPLC) was used to quantify aniracetam based on the method by Ogiso [26], with some modifications. Stock solutions of aniracetam were prepared in methanol with concentrations of 1 mg/mL. The isocratic mobile phase consisted of an aqueous phase (0.1% formic acid in purified water) and an organic phase (0.1% formic acid in acetonitrile) in a ratio of 75:25, respectively. The chromatographic separation was carried out using a Synergi 4 µm Fusion RP80Å, 150 × 4.6 mm column (Phenomenex, Auckland, New Zealand) at 25 °C and aniracetam eluted at 15 min. Flow rate was 1 mL/min, and the injection volume was 10 µL. The detection wavelength was 285 nm.

### 2.4. Preparation of Inclusion Complexes

Solid inclusion complexes of HP-β-CD and aniracetam were prepared by physical mixture for 30 min and freeze-drying methods in molar ratios of 1:1. To prepare the samples for lyophilization, aniracetam was added to HP-β-CD 50% *w*/*v* solution in a molar ratio of 1:1. The mixture was then shaken for 24 h at 200 RPM in an orbital mixture incubator (Ratek, Newcastle, Australia). Upon completion, the solution containing the aniracetam-HP-β-CD inclusion complexes was transferred into plastic vials in volumes of 2 mL. Sucrose was then added to each 2 mL sample, the amount being 20% of the total aniracetam/cyclodextrin weight. Samples were then frozen at −83 °C for >5 h. The frozen samples were then dried using a freeze drier (Labconco, Fort Scott, KS, USA) for 48 h.

### 2.5. Stability Test Using Design of Experiments

A design of experiment (DOE) approach was used to investigate changes in the stability of aniracetam under different storage conditions and with changing HP-β-CD concentrations. A two-level, full factorial design was used (MODDE 9.0, Umetrics, Umeå, Sweden). The stability factors investigated were HP-β-CD concentration, temperature, humidity, and time (Table 1). An orthogonal (balanced) model was fitted that was able to investigate the best storage conditions and the most influential terms. The design included four center points to test for reproducibility. The response measured was the concentration of aniracetam over a 12-week period. For each run sequence, a control sample of aniracetam was also included without cyclodextrin present, and subjected to the same conditions.

A constant amount of aniracetam (10 mg) was placed into 19 uncapped glass vials. HP-β-CD was then added in amounts according to the experimental matrix. The vials were then filled to 2 mL using purified water, and shaken in an orbital mixer incubator (Ratek, Newcastle, Australia) for 24 h at 200 RPM, 30 °C. For the control samples, 10 mg of aniracetam was added to 19 unrelated uncapped glass vials and made to 2 mL volume using purified water. Samples were placed into sealed boxes with differing relative humidity (RH) values (dry, 37% and 75%). Set relative humidity conditions within the closed chambers were prepared following the method described in Greenspan [27]. Saturated solutions of sodium iodide (37 ± 0.5% RH) or sodium chloride (75% RH) were added to the sealed boxes. For dry RH, silica gel was used. The relative humidity for each of the chambers was verified using a digital hydrometer (HOBO^®^, Adelaide, Australia) before sample incubation. The sealed chambers with the samples inside were then placed inside incubators with set temperatures of 0, 25, and 50 °C, in accordance with the experimental matrix. The concentration of aniracetam was quantified by RP-HPLC, as described above. Samples were diluted 1:1000 with 50:50 acetonitrile: water with 0.1% formic acid to a total volume of 1 mL.

### 2.6. Fourier-Transform Infrared Spectroscopy (FTIR)

The FTIR spectra of aniracetam, HP-β-CD, simple mixtures, and 1:1 inclusion complexes were obtained using a Varian 3100 FTIR spectrometer (Varian Inc., Palo Alto, CA, USA). All samples contained sucrose, as described above. Aniracetam, HP-β-CD, simple mixture, and inclusion complex samples were all ground and mixed thoroughly with potassium bromide (KBr) separately. Subsequent KBr disks were then prepared via compression of the mixed powder. The FTIR spectral range recorded was 500–4000 cm^−1^ with 128 scans per sample and a resolution of 2 cm^−1^, as seen in Mangolim et al. [28]. The resultant spectra were overlaid, and changes in peak assignments were recorded between constituent compounds and the inclusion complex.

### 2.7. Data Analysis

A two-tailed *t*-test was performed to identify statistically significant differences between control samples and those that included HP-β-CD (Excel 2016, Microsoft, Washington, DC, USA). *p* < 0.05 was considered statistically significant. Multiple linear regression (MLR) was used to fit interaction models (MODDE, 9.0). As the response variable distributions were skewed, they were normalized by using logarithmic transformation. The resulting models were assessed for validity using the goodness of fit (*R*^2^), goodness of prediction precision (*Q*2), and model reproducibility. Response surface plots were then created using the contour plot wizard on MODDE.

## 3. Results

### 3.1. Phase Solubility Analysis

The relationship between peak area and the concentration of aniracetam was shown by HPLC to demonstrate linear correlation over the concentration range of 5–100 μg/mL (*y* = 28762*x* + 11299). The coefficient of determination for the standard curve was *R*^2^ = 0.9998. The standard curve obtained had a CV (coefficient of variation) and bias <2%. Aniracetam displayed a characteristic single peak at approximately 12.5 min retention time with no interference from HP-β-CD at this retention time. Intraday validation CV for the three quality controls investigated (8, 30 and 70 μg /mL) was <1.2%.

**Figure 1:**
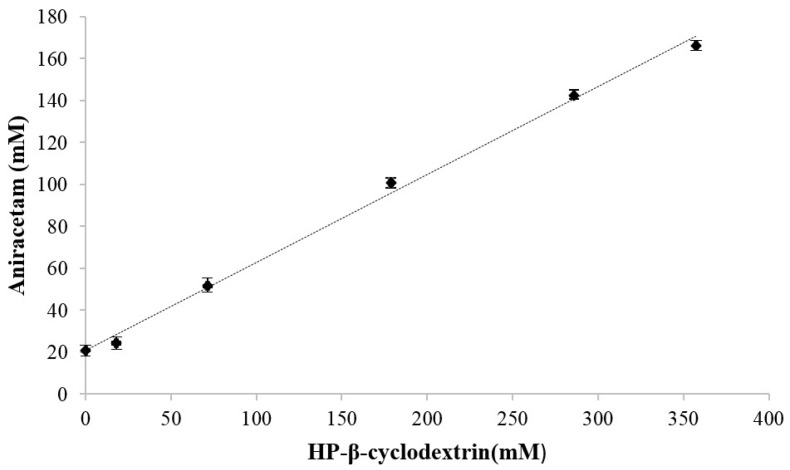
Phase solubility profile of aniracetam as a function of HP-β-CD concentration in water at 30 °C. *y* = 0.4192*x* + 20.727, *R*² = 0.9953. Each data point represents mean ± S.D, *n* = 3.

The apparent solubility of aniracetam increased as a function of increasing HP-β-CD concentration. The phase solubility profile (Figure 1) showed a linear dependence on HP-β-CD concentration. The coefficient of determination was calculated to be *R*^2^ = 0.9953 (Figure 1). The solubility of aniracetam in the absence of HP-β-CD was 20.73 mM (4.53 mg/mL) and increased to 166.22 mM (36.44 mg/mL) at a HP-β-CD concentration of 357.4 mM. The stability constant, *Kc*, was calculated to be 34 M^−1^.

### 3.2. Stability of Aniracetam:HP-β-CD Inclusion Complex

**Figure 2:**
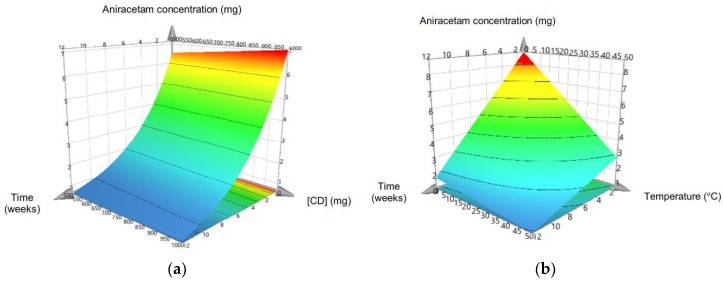
Response surface plots of remaining aniracetam concentration over time with varying (**a**) cyclodextrin concentration and (**b**) temperature over 12 weeks in different storage conditions. Plots were fitted using a multiple linear regression model in MODDE. Areas shown in red indicate storage conditions that preserved >90% of the initial aniracetam concentration.

The MLR model used was valid (*R*^2^ = 0.943, *Q*2 = 0.901, model validity 0.945) and reproducible (0.997), and so, able to be used to quantify the individual effects of the model terms on the stability of aniracetam and also synergistic interactions between variables. Temperature, time, and HP-β-CD concentration significantly influenced the degradation rate of aniracetam (Figure 2). Increasing the cyclodextrin concentration significantly (*p* < 0.05) increased the stability of aniracetam compared to aniracetam alone. The optimum stability was found with 50% *w*/*v* concentration of HP-β-CD. RH did not have a significant effect on stability over the range of 0 to 75% RH, and was a non-significant term in the MRL model (coefficient value 0.043). As temperature increased from 0–50 °C, the rate of aniracetam degradation also increased in a non-linear fashion, with temperatures below 6 °C resulting in an aniracetam concentration that was within 90% of its initial concentration (Figure 2b).

### 3.3. Characterizing Aniracetam: HP-β-CD Inclusion Complex

**Figure 3:**
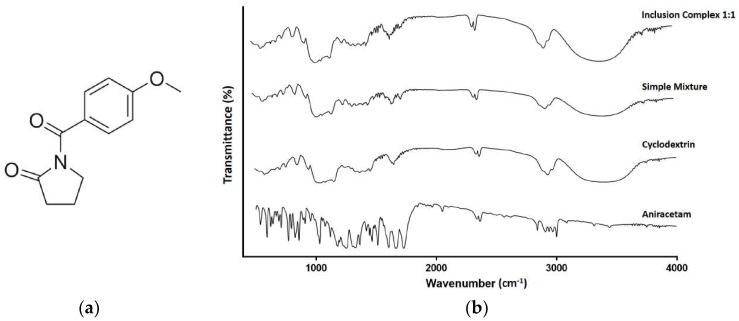
(**a**) The chemical structure of aniracetam (**b**) FTIR spectra of aniracetam, HP-β-CD, physical mixture, and inclusion complex (1:1). Scan range of 500–4000 cm^−1^, resolution of 2 cm^−1^, with 128 scans per sample.

The FTIR spectra for aniracetam and HP-β-CD (Figure 3) were characteristic of other FTIR spectra of the pure compound [28]. Key frequencies observed for HP-β-CD included 3399.59, 2929.92, 1156.32, and 1031.97 cm^−1^ (Table 2). The key frequencies for aniracetam were recorded at 1727.87, 1682.90, 1249.56, and 681.39 cm^−1^ (Table 3). Key changes in vibrational frequencies between the spectra of aniracetam and the inclusion complex, and between HP-β-CD and the inclusion complex, are displayed in Table 2 and Table 3, respectively.

Both the spectra for the inclusion complex and physical mixture are similar to the pure HP-β-CD spectrum (Figure 3b). The broad hydroxyl band of pure HP-β-CD is narrowed in the FTIR spectrum of the inclusion complex (Figure 3b). The largest observed change in frequency from HP-β-CD to the inclusion complex spectra, is the increase in *v*[O–H] frequency (Table 2). A large apparent shift to the right along the *x* axis can be observed for the *v*[OH] peaks originating at a frequency of 1031.97 cm^−1^ (Figure 3b).

There was a marked increase in the frequency of the aryl carbonyl from the spectrum of aniracetam to the inclusion complex (Figure 3b, Table 3). This increment in frequency was not shared by the carbonyl peak corresponding to the pyrrolidinone moiety, where only a very minor change in frequency was noted (Table 3). Despite the opposing changes, both carbonyl related peaks are still identifiable within the inclusion complex FTIR spectrum, being considerably flattened (Figure 3b). The greatest change that occurred for aniracetam in the inclusion complex was the increase of *v*[=C–H] stretching by +88.16 (Figure 3b, Table 3). An additional peak can be observed at a frequency of 1245.37 cm^−1^ on the inclusion complex spectrum, which is absent from the pure HP-β-CD spectrum (Figure 3b).

## 4. Discussion

The addition of HP-β-CD was able to increase the solubility of aniracetam by 819% with respect to its pure aniracetam counterpart. The total amount of aniracetam in solution increased linearly with successively higher concentrations of HP-β-CD added, and thus, the phase solubility profile can be considered an A_L_-type diagram [29]. An A_L_-type diagram means that all complexes formed are of the first order, suggesting 1:1 complexation between aniracetam and HP-β-CD occurred. The 1:1 molar ratio is also supported by the slope of the profile being less than one [29]. Increasing the solubility of aniracetam to 36.44 mg/mL, using HP-β-CD 50% *w*/*v*, enables sufficient parenteral dosing of aniracetam in animal models [29]. The linear relationship in solubility enhancement that is possible with cyclodextrin complexes gives cyclodextrins a competitive edge over co-solvents in enhancing solubility because precipitation becomes highly unlikely after oral or intravenous dosing, a trait that limits co-solvents due to their log-linear relationship [18]. Knowledge of the 1:1 molar ratio is important to consider during formulation preparation as mixtures with exceeding ratios will result in precipitation [30] that should be avoided for parenteral formulations. Recently, Saokham et al. [31] have recommended that for parenteral solutions, and specifically, for formulations designed for IV administration, excess cyclodextrin should be added to ensure that the solubilization is sufficient.

The intercept of the phase solubility profile highlighted a discrepancy within the literature concerning the aqueous solubility of aniracetam. In the present study, the solubility of aniracetam alone was 4.53 mg/mL. This is a significant increase from 0.109 mg/mL, which is reported in the literature [16]. The marked increase in apparent solubility may be explained by the limited samples used within the phase solubility analysis at lower HP-β-CD concentrations. Mayersohn et al. [16] provide the only record of aniracetam solubility, and hence, a consistent value of the solubility of aniracetam is lacking from the literature. Despite the conflicting values, insufficient parenteral solubility characteristics remain a constant.

On the basis of a 1:1 stoichiometric ratio, the stability constant was shown to be 34 M^−1^ using the phase solubility profile. The low stability constant value implies a very weak association between the two molecules, even though the data confirm a direct interaction between aniracetam and HP-β-CD exists [30]. Rapid dissociation kinetics results in the formation of a highly-accessible drug depot within the cyclodextrin complex at the site of administration [32]. Thus, free aniracetam should be absorbed rapidly at extravascular sites, with little to no absorption of the large cyclodextrin molecule. Despite the proposed advantageous properties of rapid dissociation in vivo, the literature proposes benefits for inclusion complexes with large stability constants. Cyclodextrin complexes with high stability constants have been shown to confer increased retention of therapeutic efficacy of the free drug [29].

It is known that aniracetam degrades by hydrolysis to two separate degradation products via the two amide functional groups [33]. This study investigated the nature of stability enhancement that involvement of HP-β-CD may confer on aniracetam. The hypothesis that HP-β-CD would improve the stability of aniracetam was confirmed by the results obtained in the DOE stability test. The MLR model showed that the positive relationship was consistent across all time and temperature input variables. The relationship was of statistical significance, marked as a significant model term coefficient. The optimal storage conditions to preserve the integrity of the formulation and prolong shelf-life were determined to be temperature = 0 °C, time = 1 week, relative humidity = 0%, and an HP-β-CD concentration of 50% *w/v*. The findings were consistent with the literature, giving similar results to those for other HP-β-CD inclusion complex stability studies [34,35]. The decrease in degradation may be explained by the mechanical encapsulation of aniracetam within the protective inner cavity of HP-β-CD.

The hypothesis that the degradation of the aniracetam-HP-β-CD complex would occur more readily with increasing relative humidity was shown to be false in this study. Although there was evidence to suggest that degradation occurred less at lower levels of relative humidity, it was not recognized as a significant model term coefficient. The same principle was demonstrated in Paramera et al. [36], where HP-β-CD enhanced the stability of curcumin, especially at a lower relative humidity, but to a significantly low degree. This was an interesting finding considering the known mechanism of action for the degradation of aniracetam is hydrolysis [33]. Intuitively, more exposure to water molecules would increase the relative probability of aniracetam undergoing hydrolysis at either of the two susceptible amide bonds. However, it must be noted that the formulation being tested already had the aniracetam inclusion complex present in an aqueous solvent. Water molecules were in direct contact with the complex, saturating aniracetam. Therefore, it is highly unlikely that any alterations in the relative humidity of the storage environment would act to further ‘wet’ the complex, driving hydrolysis.

FTIR has been used successfully in the past to characterize cyclodextrin inclusion complexes [24,37]. In the present study, the FTIR spectrum for HP-β-CD has good similarity to the spectrum produced by the inclusion complex, and this is often recorded in similar characterization studies using cyclodextrins as host molecules [37]. The spectrum of the simple mixture observed in the present study appears to be an almost direct superimposition of the aniracetam and HP-β-CD spectra. Characteristic peaks of both constituent molecules can be clearly observed with no significant changes in vibrational frequencies noted. The absence of change implies minimal-to-no activity of driving forces or chemical interactions between the molecules exist. It is highly unlikely that aniracetam is inserted into the cavity of HP-β-CD using a simple mixture, as the density of the central electron cloud failed to increase. Also consistent with the literature was the narrowing of the hydroxyl band at 3399.59 cm^−1^ in the inclusion complex spectrum relative to the pure HP-β-CD spectrum. Sambasevam et al. [37] suggest that the narrowing of the broad hydroxyl band of the pure HP-β-CD is an indication of the formation of the inclusion complex. The largest increment in frequency observed was attributed to the stretching of *v*[=C-H], representing the benzene moiety of aniracetam. This suggests that the benzene moiety of aniracetam was inserted into the electron-rich cavity of HP-β-CD following complexation. The finding is consistent with the literature where it is typical of aromatic moieties to display the largest increments in frequency changes, often due to their inherent hydrophobic nature being encapsulated, with priority, by the cyclodextrin [37]. The peak associated with the aryl carbonyl also displayed a significant increase in frequency. Similar to the finding of the benzene moiety, it is likely that this portion of the aniracetam molecule is also inserted and encapsulated within the hydrophobic microenvironment of HP-β-CD. The carbonyl peak corresponding to the pyrrolidinone moiety did not demonstrate any significant observable changes in frequency. Although inconclusive, this observation suggests that the pyrrolidinone moiety is not fitted within the HP-β-CD cavity, but instead, is protruding above the torus structure, exposed to the outside environment. The decreases in vibrational frequencies can be explained by the formation of hydrogen bonding and the presence of van der Waals forces during complexation of the constituent molecules [38]. Further characterization studies are needed before definitive speculation of the final structure can be made with confidence.

## 5. Conclusions

In this study, a novel parenteral formulation of aniracetam in HP-β-CD 50% *w*/*v* was designed with a significant improvement in aqueous solubility. Using this formulation, aniracetam will no longer be restricted to the oral route of administration. In turn, the limitation of poor bioavailability as a consequence of oral dosing can be avoided, offering significantly improved systemic concentrations. It is hoped that the formulation may prove useful in future research to better understand the pharmacology of aniracetam, and aid in the discovery of clinical indications pertaining to the treatment of cognitive impairment.

## Figures and Tables

**Table 1:** Factor settings for the stability conditions used in the design of experiments.

Name	Units	Type	Settings	Precision
Temperature	°C	Quantitative	0 to 50	1.25
Humidity	%	Quantitative	0 to 75	1.88
Time	Week	Quantitative	0 to 12	0.275
CD Content	mg	Quantitative	500 to 1000	12.5

**Table 2:** Comparison between the FTIR spectra intensity of HP-β-CD and the inclusion complex.

Functional Group	Wavenumber (cm^−1^)		Change Δδ
	HP-β-CD	Inclusion Complex	
*v*[OH] symmetric and antisymmetric	3399.59	3407.45	+7.86
*v*[C–H_2_]	2929.92	2926.11	−3.81
*v*[C–C]	1156.32	1155.38	−0.94
*v*[O–H] bending vibration	1031.97	1070.10	+38.13

**Table 3:** Comparison between the FTIR spectra intensity of aniracetam and the inclusion complex.

Functional Group	Wavenumber (cm^−1^)		Change Δδ
	Aniracetam	Inclusion Complex	
*v*[=C-H]	681.39	769.55	+88.16
*v*[C=O] (aryl)	1727.87	1761.22	+33.35
*v*[C=O]	1682.90	1682.82	−0.08
*v*[C-N]	1249.56	1245.37	−3.63
